# Systematic scoping review protocol of Stroke Patient and Stakeholder Engagement (SPSE)

**DOI:** 10.1186/s13643-023-02347-6

**Published:** 2023-09-30

**Authors:** Juliet Roudini, Sarah Weschke, Torsten Rackoll, Ulrich Dirnagl, Gordon Guyatt, Hamidreza Khankeh

**Affiliations:** 1 QUEST Center for Responsible Research, Berlin Institute of Health at Charité, Berlin, Germany; 2 Department of Experimental Neurology, Charité Universitätsmedizin Berlin, Berlin, Germany; 3 Department of Health Research Methods, Evidence, and Impact, McMaster University, Hamilton, ON Canada; 4 Department of Emergency and Disaster Health, University of Social Welfare and Rehabilitation Science, Tehran, Iran; 5 Department of Clinical Science and Education, Karolinska Institute, Stockholm, Sweden

## Abstract

This protocol describes a systematic scoping review of Stroke Patient and Stakeholder Engagement (SPSE), concepts, definitions, models, implementation strategies, indicators, or frameworks. The active engagement of patients and other stakeholders is increasingly acknowledged as essential to patient-centered research to answer questions of importance to patients and their caregivers. Stroke is a debilitating, long-lasting burden for individuals, their families, and healthcare professionals. They require rehabilitation services, health care system assistance, and social support. Their difficulties are unique and require the continued involvement of all parties involved. Understanding SPSE in research is fundamental to healthcare planning and extends the role of patients and stakeholders beyond that of the study subject. We will conduct a systematic literature search to identify the types of existing evidence related to SPSE, implementation strategies, indicators, or frameworks related to Patient and Stakeholder Engagement (PSE); clarify key concepts, definitions, and components of SPSE; compile experiences and prerequisites; and identify stroke research internationally. Two independent reviewers will extract data from selected studies onto a customized extraction form that has already been piloted. We integrate existing knowledge to address gaps in the literature on SPSE research by presenting the model, implementation strategies, indicators, and frameworks for stroke patients. We hope that these findings will offer future researchers a clear picture and conceptual model of SPSE.

## Introduction

Patient engagement is defined as “The active, meaningful, and collaborative interaction between patients and researchers across all stages of the research process, where research decision-making is guided by patients’ contributions as partners, recognizing their specific experiences, values, and expertise” [1]. This can include patients, other relevant stakeholders like family members, and formal and informal healthcare providers [2]. Patients and Stakeholders Engagement (PSE) can provide a unique perspective, sometimes with firsthand knowledge and experience that is more relevant and approachable to the needs of patients and stakeholders [3–5]. Community-based participatory research (CBPR) studies indicate that patient participation in research is essential for reaching unreachable or otherwise underserved patients [6, 7]. Numerous authors assert that research participation is crucial for empowering patients in their healthcare through self-empowerment, enhanced self-confidence, a sense of being respected, an effect on their mental health, the desire to contribute, and a desire to serve the community (citizenship literacy) [8–10]. Researchers also claim that by engaging patients and caregivers early in the research process, they can effectively serve as early ambassadors of research efforts and additional findings, potentially broadening audiences beyond peer-reviewed journals and facilitating the acceptance and implementation of results in the community and healthcare setting [11, 12]. Politically speaking, the majority of patients and researchers concurred that patient participation in the study process boosted credibility, which helps democratic ideals of accountability, transparency, and legitimacy in public and private organizations [4, 13]. The eminent quote “nothing about us, without us” emphasized that researchers would have a moral obligation to engage patients in research beyond the role of subjects [14]. As a result, patients and other stakeholders have a basic right to participate in the research process and should have the chance to influence its direction [15].

Although there are currently a large number of papers describing PSE activities and several models and recommendations with a wide range of quality and varied focuses, there is still a lack of consensus throughout the studies, on how to best build and foster PSE [16–18]. The gaps include an absence of understanding of how to engage a broad range of stakeholders across the healthcare system, which methods, models, and strategies of PSE are practicable throughout the earlier and later phases of research; how the opinions of stakeholders are synthesized and used to form research design, implementation, and dissemination; and how to collaborate with stakeholders to increase intervention effects, decrease disparities, and/or sustain tested interventions [19]. According to Esmail and her colleagues, research generally did not make explicit their aims for PSE to establish a cohesive, generalizable evidence base that enables comparability across studies and countries. While technique measurements of engagement were emphasized more than results, there is still a need to understand who should be engaged, when, and how. According to their studies, PSE has to develop more conceptual direction and consensus to guide evaluation processes [20]. Workman proposed developing measurable effect indicators and procedures in addition to those focusing on changes in the attitudes and degrees of satisfaction of research participants [21].

Stroke is the world’s second leading cause of mortality and lifelong disability [22]. The aged population in Europe is expected to grow by 35% between 2017 and 2050, resulting in an increase in stroke survivors due to an aging population and increasing survival rates [23]. Twenty to 50% of persons may have at least one of these challenges, such as post-stroke depression (PSD), anxiety, vascular cognitive impairment (VCI), and post-stroke fatigue (PSF), which can delay recovery and result in poor functional results and reduced quality of life [24]. However, acute treatment and prevention methods have improved over the last several decades, and the incidence of stroke continues to fall at a constant rate in Oxfordshire and other high-income countries, even though the total number of stroke incidents in aging populations is increasing [25].

Fortunately, there is overwhelming evidence that stroke is mostly preventable, curable, and manageable, with the potential to greatly lessen the burden of stroke and its long-term consequences. Nonetheless, all parties, including government agencies, scientific and stroke support groups, healthcare professionals, clinical and preclinical researchers, patients, and their families, must work together to achieve this [26]. The study by McKevitt and colleagues showed a lack of European understanding of stroke research. According to the European Stroke Action Plan for 2018 to 2030, researchers, healthcare professionals, and patient groups must enhance how research results are related to patient populations and study participants [26, 27].

Stroke patients present unique challenges to PSE, emphasizing the need for a disease-specific approach: many survivors are elderly, and they frequently have severe disabilities, including speech problems, making participation in PSE activities difficult, as opposed to, say, cancer or other similar diseases. As a result, we believe that specific prerequisites are required for successful and meaningful PSE in this field of study. Furthermore, the perspective of relatives and caregivers may be even more important than in other diseases because they bear a major fraction of the patient’s burden, but from a very different perspective. Patients’ and related stakeholders’ involvement at the start of the clinical development process can significantly improve study design and delivery. It not only allows for a better understanding of the patient’s needs, but it also allows for protocols to be considered in real-life scenarios, issues to be identified, and problems to be resolved before a study opens for enrollment. As a result of better study design, participant recruitment, retention, and protocol adherence, PSE can improve the relevance and quality of research projects.

To our knowledge, no systematic literature reviews have been conducted to describe how patient engagement has been approached and assessed in stroke research. However, there is a protocol that sounds similar to the title of our study but differs in content [28]. The two investigations have different objectives. Hall and colleagues’ review identifies and describes patient and public involvement (PPI) activities, study types, and PPI participants in published stroke research, whereas our protocol presents a thorough scoping review of Stroke Patient and Stakeholder Engagement (SPSE) concepts, definitions, models, implementation strategies, indicators, or frameworks. Both of these studies produced their protocol at different times. Furthermore, in the Hall study, data will be extracted using Joanna Briggs Institute protocols, with results collated and matched to the research cycle stage/s, but we will use Arksey and O’Malley’s six stages.

As a result, our preliminary findings reveal a lack of scientific evidence to involve all stakeholders in research on treatment and rehabilitation for this growing and vulnerable patient population. The concept of interest is thus to identify the types of evidence associated with SPSE, the extraction of experiences and recommendations, the clarification of key concepts, definitions, and components, and the identification of models, implementation strategies, indicators, or frameworks for establishing an SPSE. This protocol describes a systematic scoping review of Stroke Patient and Stakeholder Engagement (SPSE), concepts, definitions, models, implementation strategies, indicators, or frameworks at the Charité on QUEST Center for Responsible Research and the NeuroCure Clinical Research Center (NCRC) at the Berlin Institute of Health (BIH).

## Method

To accomplish the study’s objective, a comprehensive systematic scoping review will be conducted. Systematic scoping reviews are fundamentally undertaken to map certain knowledge fields. A scoping review’s objectives may include (one or more of the following): mapping key concepts within a knowledge domain, refining the definitions, and determining the limits of the knowledge domain [29]. Therefore, scoping reviews aid in identifying essential concepts and knowledge gaps, as well as addressing thorough inquiries, which may involve a variety of approaches but do not involve quality evaluation. As a result, scoping review studies aid in identifying key concepts and knowledge gaps, as well as addressing in-depth queries that may employ several methodologies but do not include a quality evaluation. We will stick to Arksey and O’Malley’s six-step structure [29] which has since received widespread support and promotion in methodological literature [30, 31].

### Identifying the research question

Due to the topic’s novelty, a scoping review will be employed to achieve the following objectives: (1) identifying the sorts of current SPSE evidence, models, or strategies for establishing SPSE; (2) clarifying the main concepts, definitions, and components of SPSE; and (3) compiling the experiences, prerequisites, or suggestions for adopting or applying SPSE.

We addressed the following questions concerning research related to stroke:

1. What are the key concepts, definitions, components, models, implementation strategies, indicators, or frameworks for establishing an SPSE?
2. How is it defined within this discipline?

### Identifying relevant studies

We will apply the following search terms to search PubMed, Web of Science, MEDLINE, EMBASE, Scopus, PsycINFO via Ovid, Science Citation Index, Cochrane Database of Systematic Reviews, Global Health, and WHO Global Health Library: stroke, patient engagement, Community-Based Participatory Research Program (CBPR), Patient and Public Involvement (PPI), citizen science, the key concepts, definitions, components, and identify models and implementation strategies, i.e., internationally.

### Study selection

This scoping review will include observational and interventional research, theories, conceptual frameworks, models, systematic reviews, and scoping reviews, as well as experimental and quasi-experimental study designs, randomized controlled trials, non-randomized controlled trials, before-and-after studies, and interrupted time-series studies. Analytical observational research, including prospective and retrospective cohort studies, case–control studies, and analytical cross-sectional studies, will also be evaluated for inclusion. Case series, individual case reports, and descriptive cross-sectional studies are also regarded as descriptive observational study designs. In addition to phenomenology, grounded theory, ethnography, qualitative content analysis, and action research, qualitative studies may utilize additional methodologies. Text and opinion pieces will also be included in this scoping review. Two reviewers will extract data from selected studies independently using a piloted, personalized extraction form. This will help to shape the development of a tailored search strategy for each information source (second phase). The references of the papers included in the review will be reviewed for additional publications in the third step of the search. Articles having titles and abstracts in English are included. This review will look at studies including stroke patients of any age, gender, or health status, as well as their family caregivers. Articles pertaining to stroke patients or rehabilitated patients with research competence in PSE will be considered for inclusion in this review.

### Table 1 inclusion and exclusion criteria

**Table 1:** Systematic scoping review protocol of models, implementation strategies, indicators and frameworks for patient and stakeholder engagement in stroke patients

*Criterion*	*Definition*
Population	The study includes past or present stroke patients (of any age, gender, or health status, as well as their family carers) their formal and informal caregivers, patient representatives, researchers, and participants from various nations
Primary research	PSE are active research participants, as opposed to passive research participants (subjects) or active clinical care recipients
Screening procedure	This scoping review defines important ideas, definitions, and components and identifies models, implementation techniques, indicators, and frameworks for the establishment of an active SPSE
Disease definitions	The American Heart Association/American Stroke Association defines a stroke as one that includes silent infarctions (including cerebral, spinal, and retinal) and silent hemorrhages [32]
Setting	Our aim will be worldwide, encompassing all nations
Study design	We will consider observational and interventional investigations, including experimental, quasi-experimental, analytical, descriptive, qualitative, systematic reviews, randomized controlled trials, non-randomized controlled trials, before-and-after studies, and interrupted time-series studies
Result and conclusion	By presenting the model, implementation strategies, indicators, and frameworks for stroke patients, we synthesize existing knowledge to address gaps in the literature on SPSE research

Articles from 1996 will be included because the expansion of public involvement in the UK began with the establishment of INVOLVE in 1996. However, the emphasis on research for patient engagement came later. After discovering that individual and community stakeholders determine critical parts of healthcare services and research, the British National Institute of Health launched the INVOLVE project to achieve this engagement [33].

### Charting the data

The review process encompasses a comprehensive assessment of the title, abstract, and full content of identified studies. The selection of papers that align with eligibility criteria will be determined by a consensus reached among reviewers during the title and abstract review phase. Two independent reviewers (JR and TR) will conduct a meticulous analysis of all titles and abstracts. Discrepancies, if any, will be resolved through the involvement of HRKH to ensure accuracy and consistency. The subsequent phase will involve a thorough examination of studies that specifically detail the involvement of stroke patients and other stakeholders in research activities. These selected studies will provide valuable models, implementation protocols, indicators, and frameworks essential for the development of Stroke Patient and Stakeholder Engagement (SPSE) initiatives. Moreover, these studies will elucidate foundational concepts, definitions, and components pertinent to SPSE.

The following information will be systematically charted for each included study:

- Author(s)
- Year of publication
- Origin/country of origin (where the study was conducted or published)
- Aims/purpose of the study
- Study population and sample size (if applicable)
- Methodology/methods employed
- Type and duration of intervention, comparator, and outcome measures (if applicable)
- Key findings relevant to the research question(s)

By amalgamating data from various sources, this systematic scoping review will provide a comprehensive overview, which enhances the precision and validity of conclusions drawn. This process is pivotal in distilling overarching patterns and discerning discrepancies within the collected evidence. The synthesized data subsequently aids in addressing research objectives, highlighting consistencies or contradictions, and offering valuable guidance for future research directions.

### Consultation

We will conduct a literature review to systematically synthesize existing knowledge and address gaps in the literature on SPSE in research by elucidating key concepts, definitions, and components, as well as identifying models, implementation strategies, indicators, or frameworks for establishing an SPSE model or strategy to support our PSE efforts to inform best practice methods for stroke patients. The results will provide a clear picture of SPSE for future research, and the study’s completion will provide SPSE researchers with a conceptual framework. The scoping review findings will be presented, discussed, and interpreted at a workshop for the multidisciplinary research team. The identification of research gaps in our study was based on two main sources: the literature review, which was limited to identifying areas of overall weakness within the field by comparing explored studies; and the consultation of researchers for patient and stakeholder engagement, which proved invaluable in identifying current issues facing patients, their stakeholders, and researchers that remained unstudied. We will identify PSE activities in this study to improve the findings and make them more helpful to policymakers, practitioners, and service providers, as well as to inform and validate the findings. The findings will be utilized in the process of developing a future research agenda for the SPSE.

### Dissemination

Our findings will be published in a peer-reviewed journal and extensively disseminated through our research groups and news media tactics in compliance with the reporting requirements for scoping reviews (PRISMA-ScR)52. 

## Discussion

Although attitudes toward research are changing, many researchers regard discussing research with patients as an unusual practice; however, some patients are motivated to participate. Furthermore, PSE has been demanded in recent years by funders, patient organizations, and others, but information and criteria, as well as clear conceptions, are inadequate. From our perspective and other studies, if the research topic is drawn from the patient's perspectives and requirements, research can be meaningful and applicable. This study will support the development of a comprehensive guideline to incorporate all stakeholders of stroke research in the research process, as they should be able to influence its direction. Based on the results of many studies, we must determine patients’ and caregivers’ points of view because sometimes a problem is a priority for us but not for the stroke patient or other stakeholders; consequently, we should try to align our priorities with theirs. We believe that these findings will provide future researchers with a comprehensive picture of SPSE and a conceptual model.
